# Characterization of the Tomato (*Solanum lycopersicum*) Pectin Methylesterases: Evolution, Activity of Isoforms and Expression During Fruit Ripening

**DOI:** 10.3389/fpls.2020.00238

**Published:** 2020-03-03

**Authors:** Bo Wen, Feng Zhang, Xiaozhen Wu, Huan Li

**Affiliations:** School of Horticulture, Anhui Agricultural University, Hefei, China

**Keywords:** tomato, pectin methylesterase genes, cell wall, fruit softening, ethylene, functional divergence

## Abstract

Pectin methylesterase (PME, EC 3.1.1.11) is a hydrolytic enzyme of pectin that plays multiple roles in different plant development processes and responses to biotic stress. To characterize the molecular evolution and functional divergence of the PME gene family, a genome-wide analysis of the PME gene family in the tomato was performed. In total, 57 non-redundant PME genes were identified, and these PME genes were divided into five groups based on their phylogeneny; their classification was supported by similar gene structures and domain distributions. The PME genes were found to be unevenly distributed among 12 chromosomes of the tomato. In addition, 11 segmental duplication and 11 tandem duplication events occurred in these PME genes, implying that both contributed to the expansion of the tomato PME gene family. Non-synonymous/synonymous mutation ratio analysis revealed that positive selection played a key role in the functional divergence of PME genes. Interspecific collinear analysis indicated a large divergence in the PME gene family after the divergence of monocot and dicot plants in ancient times. Gene expression pattern analysis suggested that PMEs plays roles in the different parts of the tomato plant, including the fruit. Three newly identified candidate genes (Solyc03g083360, Solyc07g071600, and Solyc12g098340) may have functions during fruit ripening. Immunoassays suggested that the tomato isoform PE1 and PE2 may change pectin structure at cell junctions, which could be associated with fruit softening. In addition, our analysis indicate that two undescribed PE isoforms might be active in leaves and fruits. This study increases our understanding of the PME gene family in the tomato and may facilitate further functional analyses to elucidate PME function, especially during fruit ripening.

## Introduction

Pectin is one of the most abundant macromolecules within the plant cell wall in both the middle lamella and primary wall. Pectin is a highly complex group of polysaccharides that can be divided into four types of pectic polysaccharides: homogalacturonan (HGA), rhamnogalacturonan I (RG-I), rhamnogalacturonan II (RG II), and xylogalacturonan (XGA). HGAs are a major pectic form and homopolymer of (1–4) α-D-GalA, and they are synthesized in the Golgi apparatus and deposited in the cell wall in a highly methylesterified form. During cell growth and development, some of these methyl groups can be removed by enzymes in the cell wall, such as pectin methylesterase (PME). PME (EC 3.1.1.11, CE8 of CAZy), also called pectinesterase, is widely present in plants and some microorganisms that possess a cell wall degradation function. PMEs can catalyze the de-esterification of methylesterified galacturonic acid residues of pectin, generating carboxyl groups and releasing free methanol in the cell wall. After de-esterification, the blocks of de-esterified HGA can be cross-linked by calcium to form a structure called “egg box,” which may stiffen the cell wall (Micheli, 2001). Otherwise, the de-esterified HGA could be cleaved by other cell wall enzymes, such as polygalacturonase (PG), which could result in cell wall loosening (Seymour et al., 1987).

According to the presence or absence of the PME inhibitor (PMEI) domain, PMEs can be classified into either Type I (with both PME and PMEI domains) or Type II (with the PME domain only) (Jolie et al., 2010). In plants, PME exists as multigene families, and different PME genes exhibit different expression specificities (Jeong et al., 2018). Genomic sequencing programs have revealed that there are 66 PME genes in *Arabidopsis thaliana* (Louvet et al., 2006), 89 in *Populus* (Geisler-Lee et al., 2006; Pelloux et al., 2007), 43 in *Oryza sativa* (Jeong et al., 2015), and 105 in *Linum usitatissimum* (Pinzon-Latorre and Deyholos, 2013). Research has revealed that PME plays multiple roles in plants, including methanol accumulation (Jolie et al., 2010), abscission (Sexton and Roberts, 1982), plant defense (Bethke et al., 2014), pollen tube growth (Bosch and Hepler, 2005), cotton fiber elongation (Qin and Zhu, 2011), cell release from the root cap (Stephenson and Hawes, 1994), plant pathogenesis (Raiola et al., 2011; Lionetti et al., 2012; Giancaspro et al., 2018), increasing ascorbic acid content (Rigano et al., 2018), plant systemic infection by the tobacco mosaic virus (Dorokhov et al., 1999; Chen and Citovsky, 2003), heat and salt tolerance (Wu et al., 2017; Yan et al., 2018), microspore development (Yue et al., 2018), and maintenance of tomato fruit tissue integrity and texture during postharvest shelf life (Tieman and Handa, 1994; Phan et al., 2007; Wen et al., 2013).

In the tomato, three PME isoforms have been isolated, which are named PE1, PE2, and PE3 (Simons and Tucker, 1999). PE2 is a fruit-specific isoform and represents a dominant isoform accumulated during fruit ripening (Tieman et al., 1992; Hall et al., 1993). Tieman et al. (1992) generated a PE2 antisense line, in which fruit tomato integrity was lost during ripening. The *Pmeu1* gene has also been successfully downregulated by antisense technology, and the aforementioned transgenic plant showed the loss of the PE1 isoform, and fruit softened faster (Phan et al., 2007). In a previous study, we generated a double antisense line. In double antisense fruit, only 10% of normal PE activity was remained and ripening associated pectin de-esterification was almost completely blocked. However, PE1/PE2 line only mimicked the phenotype of Pmeu1 as, and further change in fruit firmness was not observed. Comparing to PE1 isoform, PE2 was found to play a major role in pectin de-esterification and act on during fruit ripening (Wen et al., 2013).

In this study, a genome-wide analysis of the PME gene family of the tomato was conducted using genomic sequencing tools including the phylogenetic tree as well as motif composition, gene structure and domains, chromosome distribution, and gene duplication events. Furthermore, expression patterns of PME genes in different vegetative tissues and during fruit ripening was investigated. Using a PE1/PE2 double antisense line, the isoforms in different tomato tissues and esterification pattern changes during fruit ripening were characterized. Our results provide valuable information on PME gene evolution and function that will support future research of this gene family in plants, predominantly their role in fruit ripening.

## Materials and Methods

### Identification of PME Family Members in the Tomato Genome

The tomato protein sequence was downloaded from the phytozome (JGI^1^). To identify tomato PME candidates, hidden Markov model (HMM) analysis was used for the search. We downloaded an HMM profile of PMEs (Pfam01095) from the Pfam protein family database^2^. After removing all redundant sequences and short sequences, the output putative PME protein sequences were submitted to the Conserved Domain Database (CDD)^3^, Pfam^4^ (Pelloux et al., 2007), and Simple Modular Architecture Research Tool (SMART)^5^ (Letunic et al., 2004) to confirm the conserved PME domains. The predicted protein sequences lacking the PME domain were excluded. Finally, a reliable list of PME family genes was obtained.

### Multiple Alignment and Phylogenetic Analysis

The conserved domain sequences of PMEs derived from *A. thaliana* and tomato were used for phylogenetic analysis. Multiple sequence alignment was performed using ClustalX1.81 software with default parameters. Based on alignment, phylogenetic trees were constructed with the neighbor-joining (NJ) method using ClustalX1.81. Bootstrap analysis was performed using 1000 replicates. A phylogenetic tree including all tomato and Arabidopsis PME protein sequences was constructed from the ClustalW-aligned PME proteins using MEGA6.0 software (Tamura et al., 2013) with default parameters. Tomato PME genes were classified into different groups according to the topology of the phylogenetic tree and the classifications of PMEs in *A. thaliana*.

### Sequence Analysis, Gene Structure Analysis, and Identification of Conserved Motifs

Physical parameters of the predicted PME proteins, including the amino acid (aa) length, molecular weight (MW), and isoelectric point (pI), for each gene product was calculated using the online ExPASY tool^6^ (Gasteiger et al., 2003). Exon and intron structures of individual PME genes were analyzed using Gene Structure Display Server 2.0 (GSDS^7^) through alignment of cDNAs with their corresponding genomic DNA sequences (Guo et al., 2007). The presence of signal peptide and transmembrane domains was predicted using SignalP 4.0 and TMHMM v.2.0, respectively. We used the online MEME tool (version 4.12.0^8^) to analyze the conserved motif structures of the proteins encoded by tomato PME genes (Bailey et al., 2009) with the following parameters: any number of repetitions, maximum of 10 misfits, and an optimum motif width of 6–50 aa residues. The exon–intron structures of tomato PME genes were identified using the GSDS^9^ (Hu et al., 2015).

### Chromosomal Localization and Gene Duplication

The chromosomal positions of tomato PME genes were acquired from the tomato (*Solanum lycopersicum*) using the JGI genome browser. MapChart software (version 2.32) (Voorrips, 2002) was used for the mapping of tomato PME genes’ chromosomal positions and relative distances. Tomato PME gene duplication was confirmed based on two criteria: (a) the length of the shorter aligned sequence covered >70% of the longer sequence and (b) the similarity of the two aligned sequences was >70% (Gu et al., 2002; Yang et al., 2008). Two genes separated by five or fewer genes in a 100-kb chromosome fragment were considered as tandem duplicated genes (Wang L. et al., 2010). MCScanX software was used for collinearity, gene doubling, and tandem duplication analyses, and Circos was used for mapping.

### Calculation of Non-synonymous to Synonymous Substitutions

Duplicated PME gene pairs in the tomato genome were aligned using ClustalW. Next, the non-synonymous substitution rate (Ka) and synonymous substitution rate (Ks) values were calculated using KaKs_Calculator 2.0 software (Wang D. et al., 2010). The calculated Ka/Ks ratios were then analyzed to explore the selection pressure on each duplicated gene pair. Generally, a Ka/Ks ratio greater than, equal to, or less than 1 indicates positive (diversifying) selection, neutral evolution, or negative (purifying) selection, respectively.

### Plant Material and Ethylene Treatment

All tomato plants, *Lycopersicon esculentum* Mill cv. Ailsa Craig (S.A. Bowes, Glasshouse Crops Research Institute) wild-type along with the PE1, PE2, and double PE1as/2as antisense lines, were grown under glasshouse conditions with a cycle of 16 h light at 22°C and 8 h dark at 14°C. The plants reached maturity within 3–4 months. The fruit were tagged at anthesis (defined as the time of petal drop and fruit set) and harvested at different stages. The stages were defined as follows: immature green (IMG, 25 days after anthesis), mature green (MG, 40 days after anthesis), Breaker (B, fruit picked at first color change from green to yellow), and red ripe (B + X, fruit picked at X days after breaker). For the ethylene response experiment, 0.1% ethephon was applied to the stalk of the MG fruit. Leaves and fruit tissues were collected and frozen at −80°C until required.

### RNA Extraction and Real-Time Quantitative PCR Analysis

To examine tomato PME gene expression, tomato leaf, and fruit samples were collected from the greenhouse. Total RNA was extracted from the collected samples using Trizol Reagent (Invitrogen). Then, DNase-treated RNA was reverse transcribed using reverse transcriptase (Takara No. 6110A). Primers (Supplementary Table S1) were designed for real-time quantitative PCR (q-PCR) using Primer Premier 5 software. PME genes were used as an internal reference, and the primers for these gene were synthesized by Sangon Biotech, Co., Ltd. (Shanghai, China). q-PCR was performed using a CFX96 instrument to examine the gene expression in cDNA samples from cross-pollinated varieties at different developmental stages. Each reaction was performed in triplicate. The relative expression levels of tomato PME genes were calculated using the 2^–ΔΔ*CT*^ method (Livak and Schmittgen, 2001). The reaction mixtures (Takara, No. RR820A) contained the following in a total volume of 20 μL: 10 μL SYBR Premix Ex Taq II (2×), 2 μL template cDNA, 1 μL forward and reverse primers, and 6 μL water. PCR amplification was conducted under the following conditions: 95°C for 1.5 min, followed by 40 cycles of 95°C for 1.5 min, 95°C for 15 s, and 60°C for 30 s. This experiment was carried out in four biological replicates for each measurement.

### Extraction of Protein From Tomato Fruit

The tomato pericarp was homogenized in dH_2_O, 1:2 (w/v). The homogenate was then transferred to a 50-mL Falcon tube and spun for 20 min at approximately 3000 rpm. A further spin was sometimes necessary when the supernatant was not clear of fruit debris. The supernatant was discarded, and care was taken not to lose any debris. The pellet was then resuspended in 20 mL extraction buffer (1 M NaCl, 0.05 M NaAc), adjusted to pH 6.0, and left at 4°C, with stirring for 3 h. A further spin at 3000 rpm for 20 min was performed after the extraction, and the supernatant was adjusted to 80% saturation with ammonium sulfate (0.57 g/mL). It was ensured that ammonium sulfate was completely dissolved before the samples were placed at 4°C overnight. The precipitate (white) was spun down at 15,000 rpm for 20 min and resuspended in 5 mL dialysis buffer (0.15 M NaCl, 0.05 M NaAc, pH 6.0). A dialysis membrane (Medicell International, London, United Kingdom) was prepared by boiling the tubes in dH_2_O for 5 min. The samples were then loaded into the tubing and immersed in additional dialysis buffer and left overnight. After this overnight dialysis, the samples were ready to load into a column.

### Extraction of Protein From Tomato Leaf and Stem by Preparation of Acetone-Insoluble Solids

Tomato leaf and stem was homogenized in four volumes of acetone at −20°C, filtered through Miracloth (Calicoes, CA, United States), and washed with 10 volumes of 80% acetone at 4°C. An additional wash with 10 volumes of 100% acetone at 4°C was performed before drying the acetone-insoluble solids (AIS) in a vacuum overnight. The dried AIS were then resuspended in 20 mL extraction buffer (1 M NaCl, 0.05 M NaAc), rehomogenized (AIS were rather clumpy at this stage), adjusted to pH 6.0, and left at 4°C for 3 h, with stirring. An additional spin at 3000 rpm for 20 min was performed after the extraction, and the supernatant was adjusted to 80% saturation with ammonium sulfate (0.57 g/mL). It was ensured that the ammonium sulfate was completely dissolved before the samples were placed at 4°C overnight. The precipitate (white) was spun down at 15,000 rpm for 20 min and resuspended in 5 mL dialysis buffer (0.15 M NaCl, 0.05 M NaAc, pH 6.0). The dialysis membrane was prepared by boiling in dH_2_O for 5 min. The samples were then loaded into the tubing and immersed in additional dialysis buffer and left overnight. After this overnight dialysis, the samples were loaded into a column.

### Isoform Analysis

PE isoform separation was conducted through Bio-Rad heparin affinity chromatography. In heparin column, proteins can be specifically and reversibly adsorbed by heparins immobilized on an insoluble support. Different proteins have different affinity with heparin. The binding ability of a particular protein depends on its buffer composition, pH, flow rate, and temperature. Dissociation was carried out by increasing the ionic strength in the buffer with a continuous NaCl gradient. Then, a PE assay was carried out to profile different PE isoforms base on the PE activity and salt dependency.

The Bio-Rad system consisted of an Econo system controller model ES-1, Econo pump model EP-1, Econo UV monitor model EM-1, Econo buffer selector model EV-1, six-port sample injection valve model MV-6, diverter valve model SV-3, and fraction collector model 2128. The column, with a 5-mL bed volume, was equilibrated with buffer A (10 mM Tris–HCl, 10 mM NaCl, pH 7.5). The samples were applied in buffer A and isoforms eluted at a flow rate of 1 mL/min using a linear gradient of NaCl from 10 mM (buffer A) to 300 mM (10 mM Tris–HCl, 300 mM NaCl, pH 7.5). Fractions (75 × 2 mL) were collected and assayed for PE activity using the microtiter plate method. Then, 20 μL of each fraction was placed into wells in a 96-well microtiter plate. A total of 200 μL assay buffer with salt (0.5% citrus pectin, 2 mM Tris–HCl, 150 mM NaCl, 0.002% phenol red, pH 8.0) or without salt (0.5% citrus pectin, 2 mM Tris–HCl, 0.002% phenol red, pH 8.0) was added into each well. The plate was read on a Dynatech MR 5000 microtiter plate reader at 405 nm every 20 min for up to 5 h with 2-s shaking before each reading. Three independent assays have been done to confirm these results.

### Immunodot Assay

This assay was conducted based on the method of Willats and Knox (Willats and Knox, 1999). The nitrocellulose membrane was loaded with pectin incubated in 3% (w/v) phosphate-buffered saline (MP/PBS) for 1 h to block all binding sites and was then washed with tap water. The blocked sheet was incubated with monoclonal antibody JIM5 (diluted 20-fold using MP/PBS) for 1.5 h and then rinsed extensively with tap water. The sheet was incubated in secondary antibody [anti-rat IgG-horseradish peroxidase (HRP), Pharmacia], which had been diluted 5000-fold with MP/PBS for 1.5 h and washed with tap water. Finally, antibody binding was determined using the EC-ECL chemiluminescence detection kit for HRP (Geneflow Ltd.). Three independent assays have been done to confirm the results.

### Immunolocalization

The tomato pericarp was embedded into Steedman’s wax and cut to a thickness of 12 μm using a microtome (HM355 Microm) and collected on polylysine-coated slides. The slides were incubated in 97% (v/v) ethanol for 10 min three times and then rehydrated in 90% (v/v) ethanol for 10 min and in 50% (v/v) ethanol for another 10 min at room temperature (RT). Finally, the slides were washed in dH_2_O for 10 min. The water was changed, and the slides were washed for another 90 min.

Non-specific binding sites were blocked by incubation with 3% (w/v) milk protein in MP/PBS for at least 30 min. The slides were incubated with rat monoclonal antibody JIM5 that had been diluted fivefold in MP/PBS for at least 1 h at RT or overnight at 4°C and then washed three times with 1 × PBS with at least 5 min for each wash. The slides were incubated with a 100-fold MP/PBS-diluted secondary antibody [anti-rat-IgG (whole molecule) linked to FITC (Sigma)] for 1 h at RT. The slides were washed three times with PBS with at least 5 min for each wash. The slides were mounted using an antifade reagent Citifluor AF1 (Agar Scientific, Stansted, United Kingdom). Finally, these slides were examined and imaged using a fluorescence microscope (Leica Microsystems). Three biological repeats have been done to confirm this result.

## RESULTS

### Identification and Analysis of PME Family Genes in Tomato

To identify PME genes in the tomato (*S. lycopersicum*), HMM analysis and BLASTP were performed against the whole genome sequence. After removing repetitive sequences, all identified sequences were reserved and submitted to CDD, Pfam, and SMART to confirm the PME domains. Finally, 57 non-redundant PME proteins were obtained; this number is similar to the number of PME genes identified in *A. thaliana* (66; Arabidopsis Genome Initiative, 2000; Scheler et al., 2015). The 57 identified tomato PME gene family members encode predicted proteins ranging from 267 to 653 aa residues in length, with an average length of 483 aa, and computed molecular masses of 12.1–69.7 kDa, with an average of 47.2 kDa, respectively. In addition, all predicted PME proteins have pIs between 5.12 and 9.56. According to previous research, PMEs are encoded by a large multigene family that can be classified into two classes: Type I and Type II. All PMEs have a conserved pectin esterase domain (Pfam01095), but only Type I has a PME-inhibitory domain (Pfam04043) (Micheli, 2001). In tomato, 36 PMEs have been identified as Type I and 21 PMEs as Type II. Previous studies have found that the glycosylation of a PME protein affects its enzymatic activity and thermostability. Glycosylation site prediction showed that most PME proteins in the tomato have glycosylation sites ranging from 1 to 12 in number, except for Solyc04g080530 and Solyc07g065350. More details about these PMEs are listed in Supplementary Table S2.

### Multiple Sequence Alignment and Phylogenetic Analysis of Tomato PME Genes

Through the multiple sequence alignment using DNAMAN (version 6), five highly conserved characteristic sequence segments were identified: Region I, _GxYxE; Region II, _QAVAL; Region III, _QDTL; Region IV, _DFIFG; and Region V, _LGRPW. Furthermore, three CE-8 catalytic residues with D (aspartate) in Region IV and R (arginine) and W (tryptophan) in Region V were found to be conserved in these segments. The putative PMEs which do not contain these catalytic residues have been removed (Supplementary Figure S1).

Molecular phylogenetic analysis was conducted using the NJ method, and a phylogenetic tree was constructed using the domain sequences of the 57 tomato PMEs and 66 previously annotated *Arabidopsis* PMEs. The evolutionary history was inferred using the NJ method based on the JTT matrix-based model (Jones et al., 1992). All positions with less than 90% site coverage were eliminated. Evolutionary analyses were conducted in MEGA7 (Kumar et al., 2016). The phylogenetic analysis indicated that the tomato PME could be divided into five groups (Supplementary Figure S2). Among these proteins, proteins encoded by 36 tomato PME genes belonged to Type I, and proteins encoded by 21 tomato PME genes belonged to Type II.

### Gene Structure and Motif Composition of Tomato PME

**FIGURE 1:**
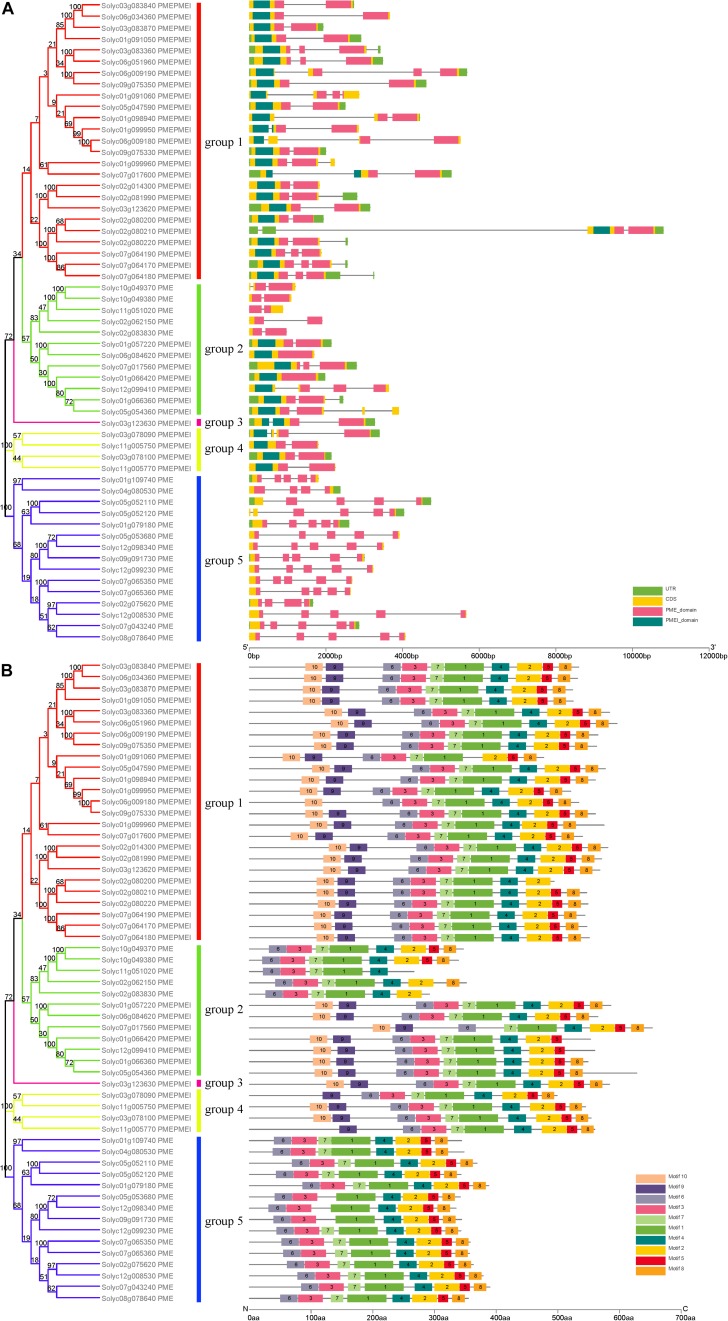
Phylogenetic relationships, conserved motif analysis, and gene structure in PME genes from tomato. **(A)** Exon–intron structure of tomato PME genes. **(B)** The conserved motif distribution of tomato PME proteins.

Evolutionary research suggests that gene structure diversity is the major force driving the evolution of gene families. To further understand the structural diversity of tomato PME genes, we analyzed the exon–intron organization of the 57 PME genes (Figure 1). The analysis revealed that PME genes in the same group usually had similar gene structures. Among tomato PME genes 2, 18, 12, 12, 12, and 1 of these genes contained one, two, three, four, five, and six exons, respectively (Figure 1A and Supplementary Table S3). Most PMEI domain located in exon 1 and most PME domain from group 1 to group 4 located in exon 1–4. Exon–intron structures of paralogous PME gene pairs were further analyzed. Among these paralogous pairs, the exon number of seven gene pairs exhibited exon–intron variations. Comparing the seven gene pairs, Soly c01g066360/Solyc05g054360, Solyc01g066360/Solyc12g099410, Solyc09g075330/Solyc01g099950, Solyc06g051960/Solyc03g0833 60, and Solyc01g109740/Solyc04g080530 gained or lost one exon, whereas Solyc06g009180/Solyc09g075330 and Solyc02 g080200/Solyc07g064170 gained or lost two exons during the long evolutionary period (Figure 1A and Supplementary Table S3). These results implied that both exon gain and loss occurred during the evolution of tomato PME genes, which may help explain the functional diversity of closely related PME genes.

Ten motifs with lengths from 6 to 50 aa residues were identified in the 57 tomato PME proteins using the MEME website (Supplementary Figure S3). Based on motif analysis, a schematic diagram representing the structure of all tomato PME proteins was constructed (Figure 1B). Most PME members in the same group had similar motif distributions. Motif 6 was found to be present in all 57 tomato PME proteins, and Motifs 1, 2, 3, 4, 5, 7, and 8 were also highly conserved in tomato PME genes (Figure 1B and Supplementary Table S4). Motifs 1, 2, 3, 4, 6, and 7 were identified to encode the PME domain, and Motif 9 and 10 were found to encode the PMEI domain, whereas the remaining motifs did not have functional annotations. Overall, the conservative motif composition and similar gene structure of PME members in the same group strongly support the reliability of the phylogenetic classification.

### Chromosomal Location and Gene Duplication Analysis

**FIGURE 2:**
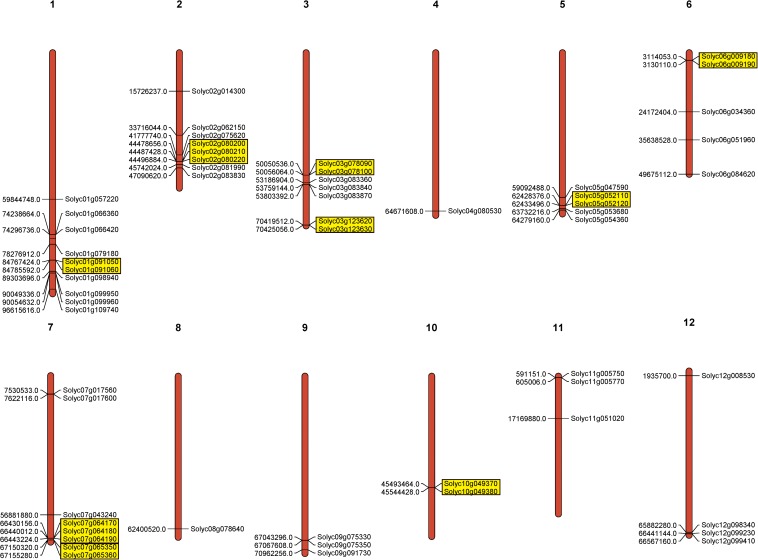
Chromosomal location of tomato PME genes. Tandem duplicated genes are marked by yellow rectangles.

To investigate PME gene chromosomal distribution in the tomato, a chromosome map was drawn according to genome annotation (Figure 2). A total of 57 PME genes were distributed in 12 tomato chromosomes. Chromosome 1 had the largest number of predicted PME genes (9), followed by Chromosomes 2 and 7 (8), and the lowest number of PMEs was found on Chromosomes 4 and 8 (1). In addition, the majority of tomato PME genes were found to be located on the proximate or the distal ends of multiple chromosomes, such as Chromosomes 1, 2, 5, and 7.

**FIGURE 3:**
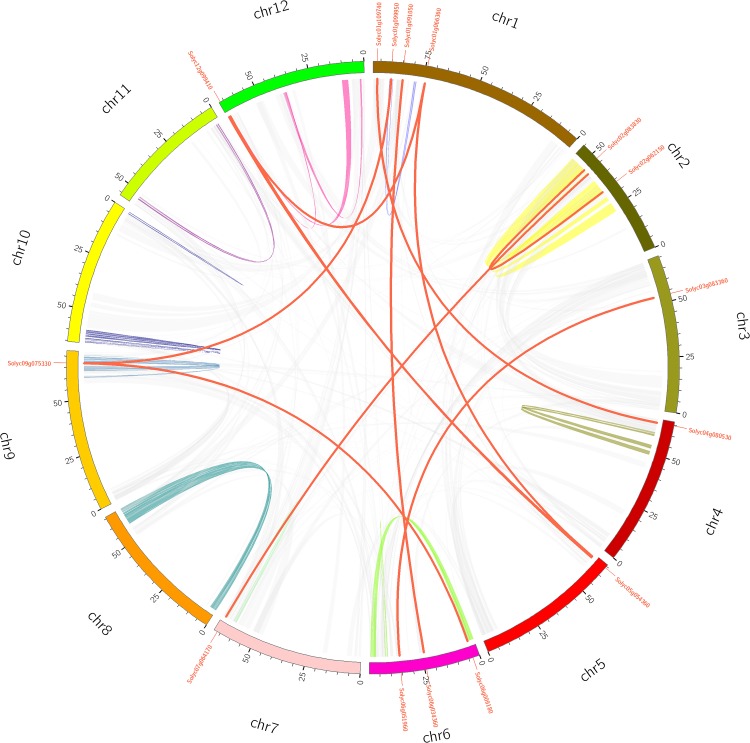
Schematic representations for the chromosomal distribution and segmental duplication of tomato. The red lines indicate segmental duplicated PME gene pairs, and gray lines and other color lines indicate all synteny blocks in the tomato genome. The chromosome number is indicated at the bottom of each chromosome.

Gene duplication events provide raw material for the generation of new genes, which in turn may facilitate the generation of new functions. Therefore, duplication events of tomato PME genes were analyzed in this study (Cannon et al., 2004). As shown in Figure 2 and Supplementary Table S5, 11 pairs of tomato PME genes (20 PME genes) were confirmed to be tandem duplicated genes. However, no tandem duplication events were identified in Chromosome 4, 8, 9, 11, and 12. In addition, 11 segmental duplication events for 18 PME genes were identified using the BLASTP and MCScanX methods (Figure 3 and Supplementary Table S5). These results indicated that both tandem duplication and segmental duplication contributed to the expansion of the tomato PME gene family. It is also interesting to see that more Type I PMEs in both segmental and tandem duplication events, which could explain the expansion of Type I PMEs in tomato genome (Supplementary Table S5). To explore the selection pressures acting on this gene family, we calculated the Ka, Ks, and Ka/Ks ratios of 22 PME gene pairs. As shown in Supplementary Table S5, the Ka/Ks values from the five pairs of tomato PME genes (Solyc02g080210/Solyc02g080220, Soly c03g078090/Solyc03g078100, Solyc03g123620/Solyc03g123630, Solyc07g064170/Solyc07g064180, and Solyc10g049370/Solyc10 g049380) were greater than 1. These results suggest these tomato PME genes experienced strong positive selective pressure during evolution, which may have caused functional divergence. The divergence time suggest most of the duplication events occurred 30–50 million years ago (Supplementary Table S5).

### Microsynteny Analysis of Tomato PME Genes

**FIGURE 4:**
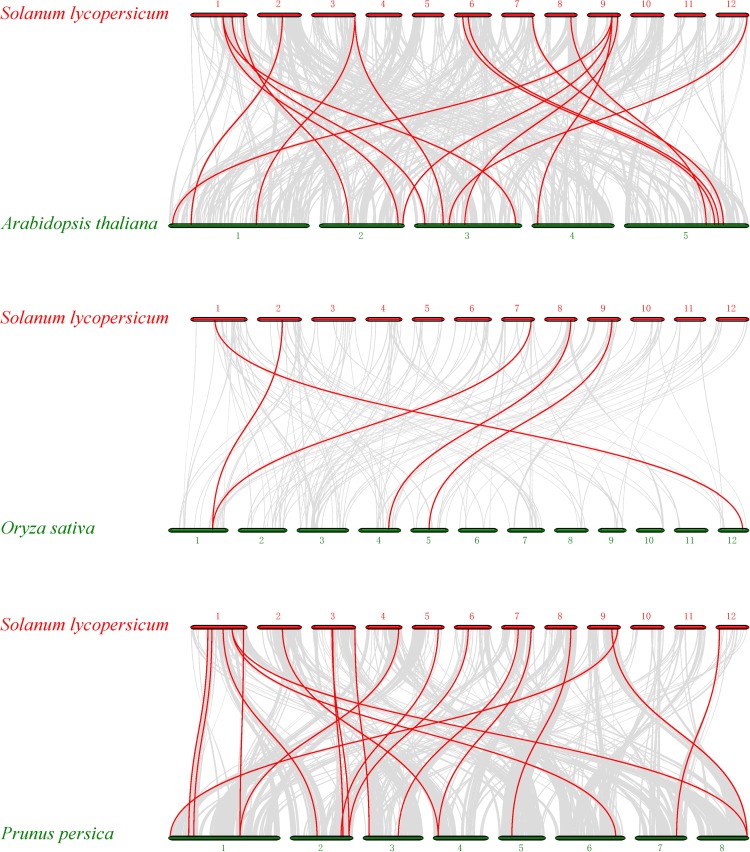
Synteny analysis of PME genes between *Solanum lycopersicum* and three representative plant species. Gray lines in the background indicate the collinear blocks within tomato and other plant genomes, whereas the red lines highlight the syntenic PME gene pairs.

To further identify orthologous genes and infer the evolution history of the tomato PME gene family, we constructed three comparative syntenic maps of tomato associated with three representative species, two dicots (Arabidopsis and peach) and one monocot (rice) (Figure 4). A total of 21 orthologous PME gene pairs were identified between tomato and peach, followed by 19 in Arabidopsis and 5 in rice (Supplementary Table S6). The total numbers of collinearity region between tomato and the three species, namely Arabidopsis, peach, and rice, were 420, 322, and 151, respectively. We found some orthologous gene pairs between tomato and Arabidopsis/peach that were not found between tomato and rice, such as Solyc01g091060/AT3G60730/Prupe.2G141200, Solyc01g099960/AT3G05620/Prupe.6G318500, Solyc01g109740/AT2G21610/Prupe.1G377100, etc. (Supplementary Table S6), indicating these orthologous pairs appeared after the divergence of dicotyledonous and monocotyledonous plants. Additionally, three tomato PME genes (Solyc02g080210, Solyc08g078640, and Solyc09g075330) were identified to have orthologous genes with all other three species, indicating that these orthologous pairs may have already existed before ancestral divergence (Supplementary Table S6). In addition, two or more PME genes from Arabidopsis matched one tomato PME gene, such as AT1G53840 and AT3G14300 orthologous to Solyc03g123620; AT1G53830 and AT3G14310 orthologous to Solyc03g123630; and AT1G02810, AT2G47550, and AT4G02330 orthologous to Solyc09g075330, implying that these genes were paralogous gene pairs. The majority of orthologous PME gene pairs had Ka/Ks values less than 1, suggesting that the PME gene family might have experienced mainly purifying selective pressure during evolution. However, positive selection also contributed to the evolution of PME genes (Supplementary Table S6).

### Identification of PME Genes Exhibiting Ripening-Associated Patterns of Expression

**FIGURE 5:**
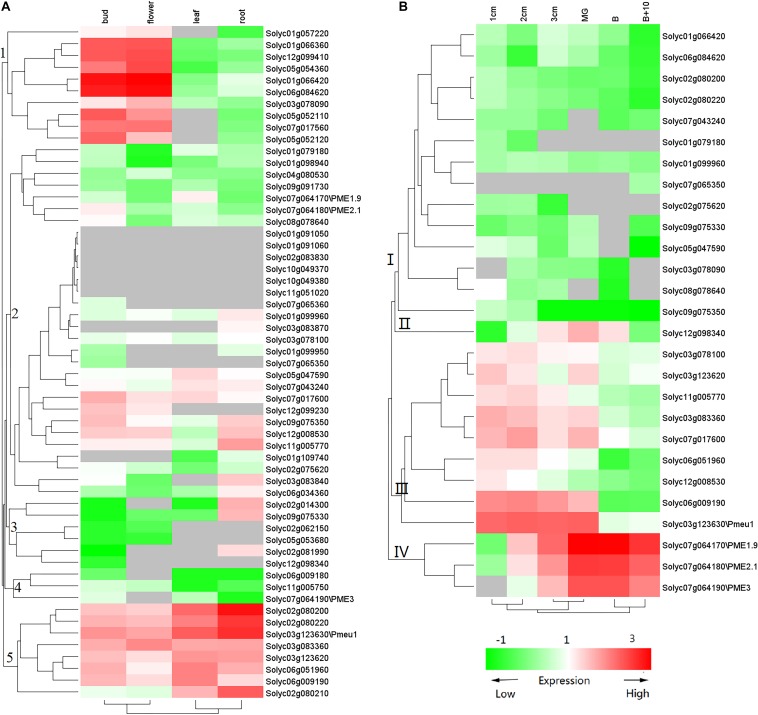
Expression profile of tomato PME genes. **(A)** PME gene expression in vegetative tissues and **(B)** PME gene expression during fruit development. The color of the cell represents transcript abundance: gray cells indicate no transcripts were detected, green-colored boxes denote low levels of expression, and red-colored boxes denote high levels of expression. This experiment was carried out in three biological replicates for each measurement.

Previous studies have suggested that PME genes play a wide range of roles during plant development, especially during fruit ripening. To gain more insights into PME gene functions, we used transcriptome data (Tomato Genome, 2012) to investigate the expression of tomato PME genes. As shown in Figure 5A, the 57 PME genes showed different expression patterns in the flower, bud, leaf, and root. Among the 57 genes, 8 PMEs in Cluster 5 showed ubiquitous expression in all vegetative tissues. In addition, ten PME genes in Cluster 1 and three PMEs (solyc02g081990, solyc02g014300, and solyc09g075330) in Cluster 3 showed the highest transcript accumulation in both the bud and flower and in the root, respectively. Among the 57 PME genes, 27 genes (RPKM > 1) were found to be expressed during fruit development (Figure 5B). Nine of the genes in Cluster 3 (solyc03g078100, solyc03g123620, solyc11g005770, solyc03g083360, solyc07g017600, solyc06g051960, solyc12g00 8530, solyc06g009190, and solyc03g123630\Pmeu1) showed the highest transcript accumulation during fruit development, three in Cluster 4 (solyc07g064170\PME1.9, solyc07g064180\PME2.1, and solyc07g064190\PME3) during fruit ripening, and one (solyc12g098340) during both fruit developmental and ripening stages. Additionally, some of duplicated gene pairs showed different expression patterns. For example, Solyc02g080200 was highly accumulated in the bud and flower, whereas its duplication gene, Solyc07g064170, was expressed at a high level during fruit ripening, suggesting subfunctionalization after a duplication event.

**FIGURE 6:**
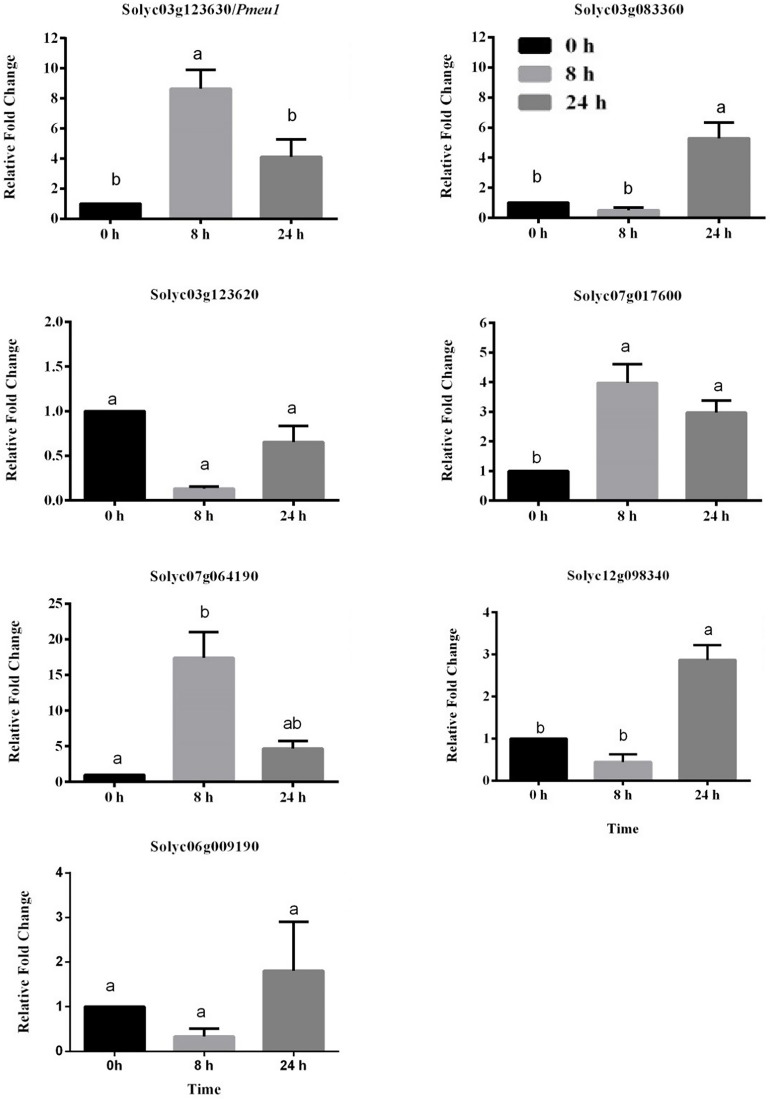
Expression profiles of seven selected tomato PME genes in response to ethylene treatment. Data are the means ± standard error (*n* = 4). Significant differences (*p* < 0.05) between means are indicated by different small letters.

To further confirm whether the expression of PME genes was influenced by ethylene, nine PME members highly expressed (RPKM > 25) in the ripening stage were selected (Solyc03g123630\Pmeu1, Solyc03g083360, Solyc03g123620, Solyc07g017600, Solyc07g064190/PME2, Solyc12g098340, and Solyc06g009190). Because the three segmental duplication genes of Solyc07g064170, Solyc07g64180, and Solyc07g64190 have very similar sequences that cannot be distinguished through qRT-PCR amplification, Solyc07g64190 is presented as a representative gene in Figure 6. It can be noticed that eight out of these nine selected genes belong to Type I PME, except Solyc12g098340 (Supplementary Table S7). Among the nine selected genes, the expression of five PME genes (Solyc03g123630\Pmeu1, Solyc03g083360, Solyc07g017600, Solyc07g064190/PME2, and Solyc12g098340) were found to be induced by ethylene. Notably, as a paralogous gene pair, Solyc03g123630\Pmeu1 responded to ethylene, but Solyc03g123620 did not, suggesting its functional diversity after a duplication event.

### PE Isoforms in Tomato Fruits, Stems, and Leaves

**FIGURE 7:**
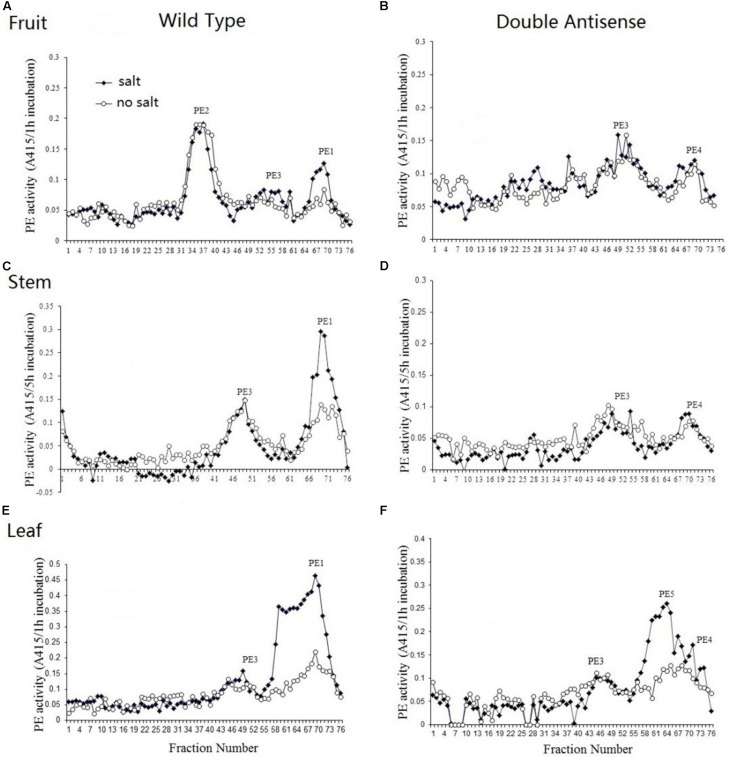
PE isoform profiles from the fruit, stem, and leaf of wild-type and PE1/PE2 double antisense plants. Total PE was extracted from the fruit, stem, and leaf of either wild-type **(A,C,E)** or PE1/PE2 double antisense **(B,D,F)**.

Total protein was extracted from the MG fruit, stem, and leaf of both wild-type and PE1/PE2 antisense plants and were then profiled by heparin column chromatography. The PE assay was conducted with and without salt (NaCl). In Figure 7A, the PE isoform profile obtained from wild-type MG fruit tissue shows three independent isoforms eluting between fractions of 31 to 43, 46 to 61, and 62 to 74, respectively. According to the nomenclature of Tucker and Grierson (1982), these correspond to PE2, PE3, and PE1, respectively. Both PE2 and PE3 activity was salt-independent, whereas PE1 was a salt-dependent isoform (Supplementary Table S7). In the fruit of double antisense plants (Figure 7B), the peak corresponding to PE2 was almost completely eliminated, PE3 was unaffected, and PE1 was suppressed to a minor peak. Notably, in the corresponding profile without salt, the PE activity of this minor peak was not reduced as it was in the corresponding peak in the wild-type fruit. As this activity might result from salt independent PE1 activity or from the activity from an unknown PE, we speculatively annotate this as PE4 (Figure 7B). Figure 7C shows that the PE activity in the wild-type stem can be resolved into two peaks. According to the elution time and salt dependency, the smaller peak eluted between fraction 41 and 56 was recognized as PE3 and the larger one eluted from fraction 62 to 76 was identified as PE1. In the double antisense stem (Figure 7D), PE3 was unaffected, and the major isoform PE1 was considerably suppressed to reveal a salt-independent peak PE4. As shown in Figure 7E, PE activity from the wild-type leaf could be resolved into one small peak representing isoform PE3 and a very large peak from fraction 55 to 76, which contains a shoulder on the left-hand side. Within the larger peak, two independent peaks were observed, indicating different salt dependencies. In the PE1/PE2 double antisense leaf, the isoform peak near fraction 69 was suppressed to smaller residual activity (Figure 7F). Combined with the elution time and salt dependency, this peak could be recognized as PE1. The shoulder peak around fractions 59 was salt-dependent and unaffected by the silencing of PE1 and PE2. As this indicates an undescribed isoform, we speculatively annotate this as PE5. Similar to the findings in both the fruit and stem, the small residual peak at fraction 69 was not influenced by salt, which may represent the activity of PE4.

### Immunodot Blot and Immunolocalization by Monoclonal Antibody JIM5

**FIGURE 8:**
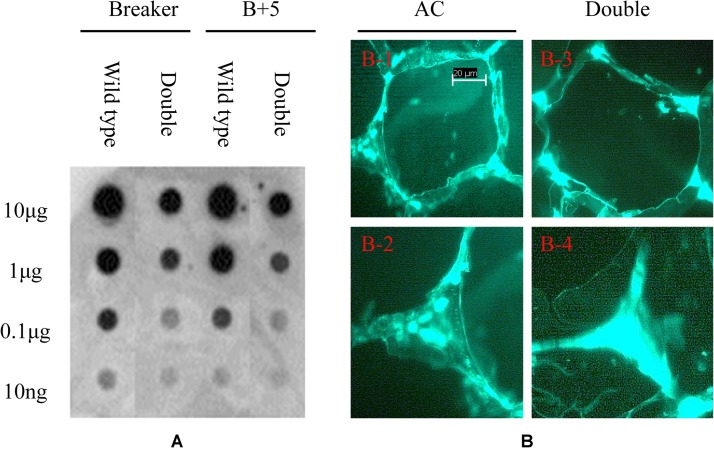
Immunodot blot and immunolocalization analysis of tomato fruit cell wall. Pectin was extracted from tomato wild-type and PE1/PE2 antisense fruit pericarp and loaded onto nitrocellulose membrane for dot blot analysis using monoclonal antibodies of JIM5 **(A)**. Immunolabeling of tomato pericarp with monoclonal antibody JIM5 **(B)**.

To detect the variation of pectin esterification, the monoclonal antibody JIM5 was used to probe the specific epitope in cell wall extracts. As shown in Figure 8A, the epitopes of JIM5 were reduced in the PE1/PE2 fruit compared with the wild-type fruit in both Breaker and B + 5 stages, which suggests that PE1 and PE2 may change the HGA esterification pattern in the cell wall during tomato fruit ripening. To determine the HGA structure change in the cell wall, the tomato pericarp from the Breaker fruit of wild-type and PE1/PE2 antisense plants was embedded in Steedman’s wax, and the embedded tissue was then sectioned and immunolocalized with the antibody JIM5. As shown in Figure 8A, JIM5 epitopes were found to be distributed across the entire wall of the wild-type fruit (Figures 8B-1,2). It seemed that, in some regions, the epitope was more prevalent and thus appeared as brighter spots. However, in the fruit of double antisense plants, JIM5 epitopes were mostly concentrated in the corners of intercellular spaces, and a very low signal was detected in other regions of the cell wall (Figures 8B-3,4). Compared with the wild-type fruit, epitope binding appeared to be much stronger and uniform in the cell corners of the fruit of PE1/PE2 antisense plants.

## Discussion

Pectin methylesterases are widely present in the plant kingdom and play multiple roles in plant development. In this study, 57 non-redundant PME genes from the tomato genome were identified. The number of PME genes in the tomato were higher than that in the monocot *O. sativa* (43) (Jeong et al., 2015) but lower than that in the dicots *Arabidopsis* (66) (Louvet et al., 2006*)*, *Populus* (89) (Geisler-Lee et al., 2006; Pelloux et al., 2007), and *L. usitatissimum* (105) (Pinzon-Latorre and Deyholos, 2013). Sequence comparison revealed that most PMEs contained five highly conserved signature sequences, in which one Asp, one Arg, and one Trp were found to be conserved and crucial for PME function, as suggested by previous studies (Markovic and Janecek, 2004; Pelloux et al., 2007). Phylogenetic analysis indicated that these PME genes could be divided into five groups; the classification is supported by the similar motif composition and gene structure in each group (Figure 1), and this finding is also consistent that for Arabidopsis in the study by Louvet et al. (2006).

Many studies have shown that structural diversification of genes plays a key role in the evolution of multigene families and in functional differentiation (Han et al., 2016). In the present study, we found that 57 PME genes contained different numbers of exons and introns, and that exon gain and loss were found in seven closely related paralogous gene pairs, which may explain why PMEs play a wide range of roles during plant development. Motif 9 and motif 10 was identified as the PMEI domain, and it was demonstrated to have a role in preventing the early demethylesterification of pectins in the Golgi apparatus (Bosch and Hepler, 2005). In the tomato phylogenetic tree, the PMEI domain was majorly restricted to Groups 1, 2, 3, and 5 PME genes, implying some specific roles in plant development (Figure 1).

In the tomato genome, 57 PME genes were unevenly located across 12 chromosomes. Gene duplication plays a critical role in the expansion of gene families (James et al., 2003; Cannon et al., 2004). It was previously found that both tandem duplication and segmental duplication were the key factors influencing the expansion of gene families (Cao et al., 2016). In this study, a total of 22 duplication events were identified for tomato PME genes. Among of them, 11 gene pairs (18 PME) involved segmental duplication, and 11 gene pairs (20 PME) involved tandem duplication. This result suggests that both segmental and tandem duplication contributed to the expansion of the tomato PME gene family.

Interspecific collinear analysis showed that the numbers of orthologous genes between tomato and peach (21) and between tomato and Arabidopsis (19) were greater than that between tomato and rice (5). This result indicated that a large divergence of the PME gene family after the divergence between monocot and dicot plants in ancient times. In theory, Ka/Ks < 1 indicates purifying or negative selection, Ka/Ks = 1 indicates neutral selection, and Ka/Ks > 1 indicates positive selection (Hurst, 2002). In this study, some paralogous and orthologous gene pairs presented Ka/Ks ratios >1, indicating that the PME gene family underwent strong positive selection pressure and tended to acquire new functions in evolution.

In this study, the expression patterns of the 57 PME genes were investigated using transcriptome data. Among them, seven genes (Solyc05g052110, Solyc07g017560, Solyc05g052120, Solyc01g066360, Solyc12g099410, Solyc01g99940, and Solyc05g054360) showed flower- and bud-specific expression, indicating key roles during flower initiation. In Arabidopsis, pollen tube growth was interrupted after one PME gene, VGD1 (At2g47040), was mutated, implying that PME is involved in the flowering process (Pina et al., 2005). Three tomato PMEs (solyc02g81990, solyc02g014300, and solyc09g075330) were found to be specifically expressed in the root, indicating that they may have a function in tomato root development. The role of PME in root elongation has been seen in other plants. For example, an *atpme3* mutant showed decreased PME activity and had a 20% reduction in root length compared with the wild-type, whereas AtPME3 overexpressors showed the opposite phenotype (Hewezi et al., 2008).

Twenty-seven PMEs were identified as fruit-development-related genes, including the previously well characterized solyc03g123630\Pmeu1 (encoding isoform PE1) as well as solyc07g064170\PME1.9, solyc07g064180\PME2.1, and solyc07g064190\PME3 (encoding isoform PE2) (Hall et al., 1993; Phan et al., 2007). Among of them, the expression of five genes (Solyc03g123630\Pmeu1, Solyc03g083360, Solyc07g071600, Solyc07g064170/Solyc07g064180/Solyc07g064190, and Solyc12 g098340) was found to be regulated by ethylene, implying they have important functions in fruit ripening. In the banana (*Musa acuminata*), PME genes also showed ethylene-dependent expression (Dominguez-Puigjaner et al., 1997). Moreover, as paralogous gene pairs, Solyc03g123630\Pmeu1 responded to ethylene, but Solyc03g123620 did not, suggesting functional differentiation after a duplication event.

According to enzymatic salt dependency, PE isoforms can be classified into two groups: salt-dependent isoforms and salt-independent isoforms (Warrilow et al., 1994; Phan et al., 2007). In the tomato fruit, the major isoforms PE1 and PE2 represent salt-dependent and salt-independent isoforms, respectively (Warrilow et al., 1994; Phan et al., 2007). In this study, PE3 was also proven to be a salt-independent isoform. Using a PE1/PE2 double antisense line, we detected persistent PME activity in the fruit that appeared to be salt-independent, and this can have multiple origins. Either PE1 has some, albeit much reduced, salt independent activity here, or there is another undescribed isoform (speculatively called PE4) which contributes in a minor way to the activity. To date, few PE-related studies have been conducted in vegetative tissue in tomato plants. Here the PE isoform profiles of both the tomato stem and leaf were obtained. The results demonstrated that PE1 is the major isoform in stems and leaves. Interestingly, the analysis of the double antisense plants revealed the contribution of an unknown isoform (speculatively called PE5) activity in leaf extracts. Gaffe et al. (Gaffe et al., 1994) observed two PE isoforms in the tomato stem, both of which showed ubiquitous expression in different tissues and different pIs, which may be equivalent to the PE1 and PE3 isoforms found in the present study. At least three, perhaps even four PME isoforms are active in the tomato fruit, which are the salt-dependent isoform PE1 and the salt independent isoforms PE2, PE3, and potentially PE4. Two or three isoforms have been identified in the tomato leaf: the salt-independent isoforms PE3 and the salt-dependent PE1 and potentially PE5. In addition, in the present study, three isoforms were identified in the tomato stem: the salt-dependent isoform PE1 and salt-independent isoforms PE3 and potentially PE4 (Supplementary Table S7). Collectively the observations here suggest that upto five different isoforms might be active across the different plant parts, and that different isoforms are activate in the fruit as compared to the leaves and stems. In addition, previous study have proved that PE1 is encoded by Pmeu1 and isoform PE2 is encoded by three tandem duplication genes of Solyc07g064170/Solyc07g064180/Solyc07g06410 (Hall et al., 1994; Phan et al., 2007). Further investigations by proteomics are required to identify the isoforms of PE3, PE4, and PE5 in future research.

Previous research revealed that only 10% of PME activity remained in the fruit of PE1/PE2 double antisense plants, and ripening-associated pectin de-esterification was almost completely blocked (Wen et al., 2013). JIM5 can recognize a partially and relatively low methyl-esterified HGA epitope (Clausen et al., 2003). As shown in Figure 8A, JIM5 epitope reduction was found after downregulating PE1 and PE2 in the tomato Breaker fruit, suggesting that PE1 or PE2 de-esterify HGA before fruit ripening. Through immunolocalization, the low esterified JIM5 epitopes were found to concentrate at cell wall corners and intercellular spaces in the fruit of PE1/PE2 antisense plants; however this phenomenon was not observed in the wild-type fruit. This cell wall structure change may be associated with the faster softening phenotype in the fruit of PE1/PE2 antisense plants (Wen et al., 2013). As mentioned by Jarvis et al. (2003), cell turgor pressure tends to separate adjacent cells. Plant cells resist this separation mainly through adhesion within a cell wall–reinforcing zone located in the cellular junction area. This area is occupied by networks of low-esterified pectic polymers and accumulates high levels of calcium. This pectic network contains at least three types of cross-links to bind the network together, which are calcium bridge links, covalent links through alkali labile esters and amides, and alkali-resistant covalent links (Guglielmino et al., 1997). Before ripening, the fruits of both PE1/PE2 antisense and wild-type plants showed similar textures (Wen et al., 2013), which could imply that at this stage, covalent links dominate cell adhesion, and calcium bridges contribute very little to it. However, with further ripening, some changes may occur in this area, and the covalent cross-links may be broken. At this point, calcium bridges in this junction area may become the major factor resisting cell separation. PE1 or PE2 could specifically produce block-wise de-esterified pectin in the junction zone that interacts with Ca^2+^ to form calcium pectate gel, resulting in fruits that soften slowly. However, when PE1 and PE2 were suppressed, the calcium bridge structure could no longer be formed. As the covalent cross-links in the network decrease during ripening, PE1/PE2 fruits tend to soften faster.

## Conclusion

In summary, a total of 57 tomato PME genes were identified and divided into five groups; the classification is supported by the exon–intron structure, conserved motif distribution, and phylogeny. Chromosomal mapping and microsynteny analysis suggested that these PME genes were unevenly distributed in all tomato chromosomes. Both segmental duplication and tandem duplication were found to contribute to PME gene expansion in the tomato genome. Ka/Ks analysis suggested both paralogous and orthologous PME gene pairs experienced strong positive selection during evolution. The gene expression pattern in various tissues suggested that PME may function in organ development and fruit ripening. Three PME genes (Solyc03g083360, Solyc07g071600, and Solyc12g098340) were identified as new candidates for fruit ripening. Immunoassay suggested that isoforms PE1 and PE2 may be involved in pectin structure modification in cell junction areas, which could be associated with tomato fruit softening. In addition, our analysis indicate that two undescribed PE isoforms might be active in leaves and fruits. This study provides useful information for further functional analysis of the PME gene family in the tomato.

## Data Availability Statement

The data generated by this study can be found in Figshare using accession number 10.6084/m9.figshare.10007573.v1.

## Author Contributions

BW conceived and designed the experiments. BW, FZ, XW, and HL performed the experiments. FZ and XW analyzed the data. FZ, XW, and HL contributed reagents, materials, and analysis tools. BW and FZ wrote the manuscript.

## Conflict of Interest

The authors declare that the research was conducted in the absence of any commercial or financial relationships that could be construed as a potential conflict of interest.

## Footnotes

1 https://phytozome.jgi.doe.gov/pz/portal.html

2 http://pfam.xfam.org/

3 https://www.ncbi.nlm.nih.gov/Structure/bwrpsb/bwrpsb.cgi

4 http://pfam.xfam.org/search

5 http://smart.embl-heidelberg.de/

6 http://web.expasy.org/compute_pi/

7 http://gsds.cbi.pku.edu.cn/

8 http://alternate.meme-suite.org/tools/meme

9 http://gsds.cbi.pku.edu.cn/
