# Supplementary material for: Characterization of the Tomato (Solanum lycopersicum) Pectin Methylesterases: Evolution, Activity of Isoforms and Expression During Fruit Ripening
Source: Front Plant Sci. 2020 Mar 3;11:238. doi: 10.3389/fpls.2020.00238 (PMC7063471; doi:10.3389/fpls.2020.00238)
Supplement: Supplementary file 8 [file Presentation_1.pdf]

Supplementary Figures

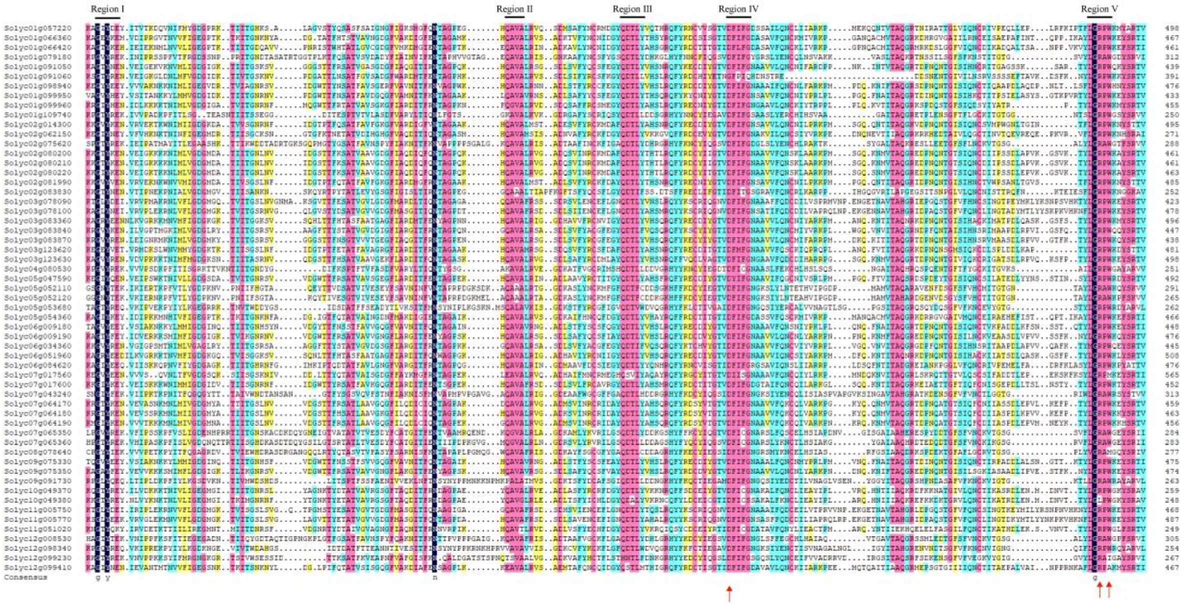

Figure S1 | Amino acid sequences alignment of conserved segments of 65 tomato PMEs. The five typical conserved segments of PMEs, referred to as Region I, II, III, IV and V. The consensus sequence is shown by letter logos. Different highlighted colors indicate different similarities, respectively (black: 100%, magenta: ≥75%, cyan: ≥ 50% and yellow: ≥ 33%).

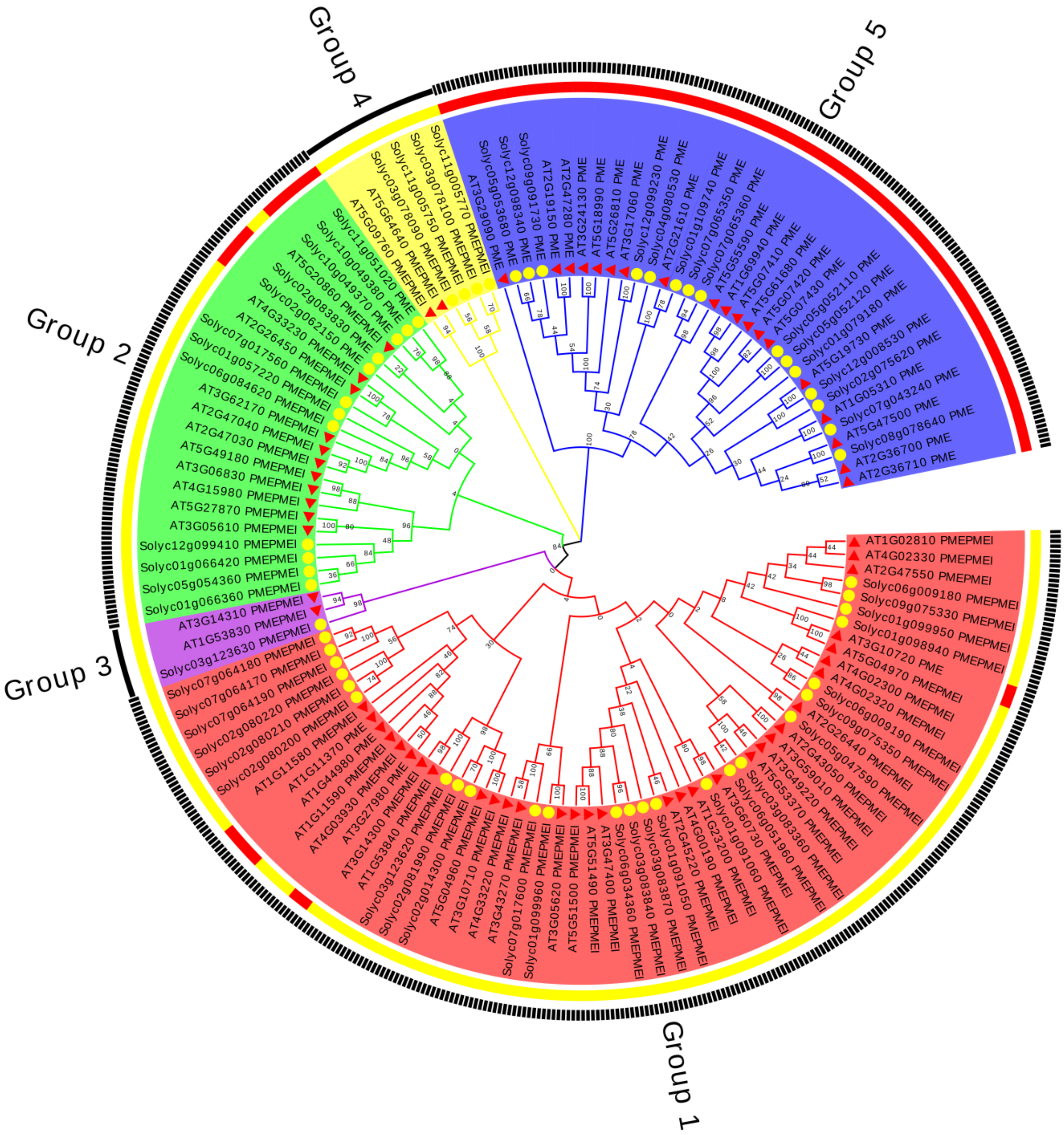

Figure S2 | Unrooted phylogenetic tree representing relationships among PME of Tomato and Arabidopsis. The different branch colors and leaf background colors indicate different groups of PME domains. The yellow circle represents tomato PME genes and the red triangle represents Arabidopsis PME genes. the yellow arc on the outer circle represents type I PME gene and the red arc represents type II PME gene.

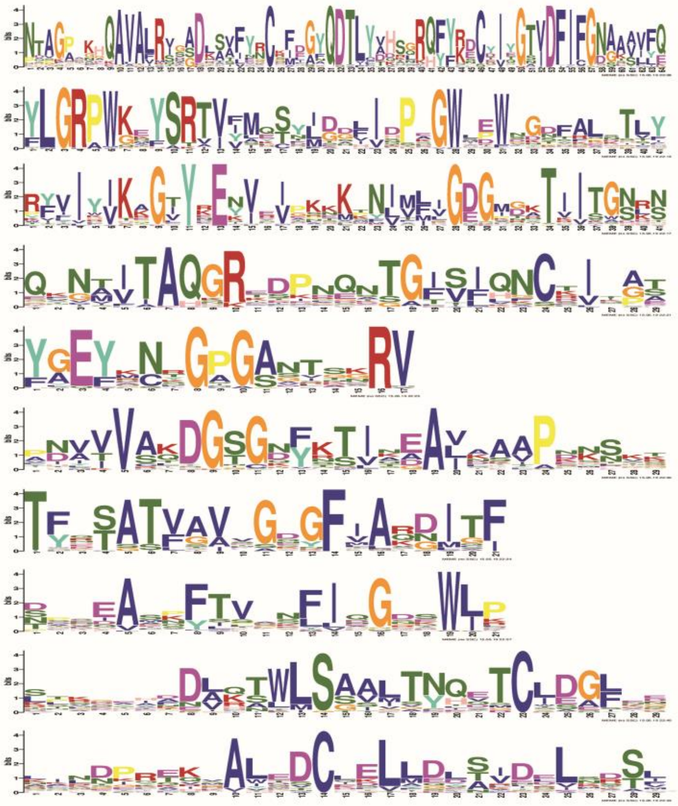

	E-value	Sites	Width
motif 1	5.4e-1917	50	64
motif 2	4.8e-1120	50	41
motif 3	2.6e-932	50	41
motif 4	7.4e-623	50	29
motif 5	2.7e-385	50	17
motif 6	1.8e-531	50	29
motif 7	1.6e-457	50	21
motif 8	6.90E-305	50	21
motif 9	8.0e-314	37	29
motif 10	3.30E-190	33	29

Figure S3 | Analysis and distribution of conserved motifs in tomato PME proteins.
